# An MRI framework for respiratory motion modelling validation

**DOI:** 10.1111/1754-9485.13175

**Published:** 2021-03-26

**Authors:** Giorgia Meschini, Chiara Paganelli, Alessandro Vai, Giulia Fontana, Silvia Molinelli, Andrea Pella, Viviana Vitolo, Amelia Barcellini, Ester Orlandi, Mario Ciocca, Marco Riboldi, Guido Baroni

**Affiliations:** ^1^ Department of Electronics, Information and Bioengineering Politecnico di Milano Milan Italy; ^2^ National Centre for Oncological Hadrontherapy (CNAO) Pavia Italy; ^3^ Department of Medical Physics Ludwig‐Maximilians‐Universität (LMU) Garching bei München Germany

**Keywords:** 4DMRI, breathing motion, MRI‐guidance, radiation oncology imaging, respiratory motion modelling

## Abstract

**Introduction:**

Respiratory motion models establish a correspondence between respiratory‐correlated (RC) 4‐dimensional (4D) imaging and respiratory surrogates, to estimate time‐resolved (TR) 3D breathing motion. To evaluate the performance of motion models on real patient data, a validation framework based on magnetic resonance imaging (MRI) is proposed, entailing the use of RC 4DMRI to build the model, and on both (i) TR 2D cine‐MRI and (ii) additional 4DMRI data for testing intra‐/inter‐fraction breathing motion variability.

**Methods:**

Repeated MRI data were acquired in 7 patients with abdominal lesions. The considered model relied on deformable image registration (DIR) for building the model and compensating for inter‐fraction baseline variations. Both 2D and 3D validation were performed, by comparing model estimations with the ground truth 2D cine‐MRI and 4DMRI respiratory phases, respectively.

**Results:**

The median DIR error was comparable to the voxel size (1.33 × 1.33 × 5 mm^3^), with higher values in the presence of large inter‐fraction motion (median value: 2.97 mm). In the 2D validation, the median estimation error on anatomical landmarks’ position resulted below 4 mm in every scenario, whereas in the 3D validation it was 1.33 mm and 4.21 mm when testing intra‐ and inter‐fraction motion, respectively. The range of motion described in the cine‐MRI was comparable to the motion of the building 4DMRI, being always above the estimation error. Overall, the model performance was dependent on DIR error, presenting reduced accuracy when inter‐fraction baseline variations occurred.

**Conclusions:**

Results suggest the potential of the proposed framework in evaluating global motion models for organ motion management in MRI‐guided radiotherapy.

## Introduction

The management of respiratory motion in external beam radiotherapy is of primary importance for tumour coverage and sparing of the surrounding healthy tissues.^1^ Different treatment solutions have been proposed such as breath‐holding, respiratory gating or tumour tracking, with image guidance as a crucial tool to plan, adapt and verify the treatment.^2^

Recently, magnetic resonance imaging (MRI) has emerged as an ideal image modality for treatment guidance, with in‐room MRI‐linacs clinically available^3^ and MRI‐guided proton therapy under investigation.^4^ Indeed, MRI allows safely repeated scans with no additional ionizing radiation, enhanced soft‐tissue contrast and fast imaging sequences.^5^, ^6^ To investigate intra‐ and inter‐fraction motion variability and cycle‐to‐cycle variations, respiratory‐correlated (RC) 4‐dimensional MRI (4DMRI) can be retrospectively reconstructed,^7^, ^8^, ^9^, ^10^, ^11^ and time‐resolved (TR) 2D cine‐MRI acquired, but limitations are still present in gaining 3D TR images with sufficient spatio‐temporal resolution.^6^, ^12^

To estimate 3D TR information of tumour and surrounding organs, respiratory motion modelling techniques have been explored from the 2D or 1D information available.^13^ This provides very useful information both for taking into account breathing variability in dose calculations^3^ and for online treatment monitoring.^14^, ^15^ Among the different motion modelling strategies, an attractive option is given by global motion models, which usually make use of deformable image registration (DIR) between 4D imaging respiratory phases to characterize non‐rigid anatomic and pathological variations and find a relationship with an external (e.g. derived from body surface motion or pressure signals) or image‐based surrogate (e.g. extracted from 2D TR images) to estimate the respiratory motion of the entire irradiated site.^13^ However, the evaluation of model performance in case of variabilities of the respiratory pattern is still a challenge due to limitations in image acquisition and data availability. Ground truth is usually not available when testing a global motion model on clinical data, and recent literature mainly relies on computational deformable phantoms.^16^, ^17^ Although obtaining the ground truth motion from clinical data is problematic, mainly due to the uncertainty of DIR techniques,^18^, ^19^ the availability of imaging data depicting ground truth respiratory states can significantly support the experimental validation of global motion models. To this aim, repeated MRI acquisitions on real patients can provide imaging data and respiratory surrogates to test, before clinical implementation, the accuracy of any respiratory motion model.

In this study, we propose an MRI‐based validation framework for the assessment of global motion models performance in MRI‐guided treatments of patients with abdominal lesions. The framework relies on repeated RC 4DMRI and TR 2D cine‐MRI being able to account for breathing motion variabilities. For our analyses, we consider a global motion model already adopted in the literature,^20^, ^21^ which we build on an RC 4DMRI and test on both TR 2D cine‐MRI and additional RC 4DMRI. The mentioned data set is therefore put forward to provide the framework for evaluating motion models’ ability in compensating for intra‐ and inter‐fraction variabilities.

## Materials and methods

### MRI‐based validation framework

The proposed framework entails two acquisition sessions (S1 and S2) on each patient, performed on different days with the same setup in order to sample inter‐fraction breathing variations. In each session, two RC 4DMRI (R1 and R2) were acquired to capture intra‐fraction breathing pattern variability. In both sessions, also a TR 2D cine‐MRI scan was performed to sample cycle‐to‐cycle breathing variations.

**Fig. 1:**
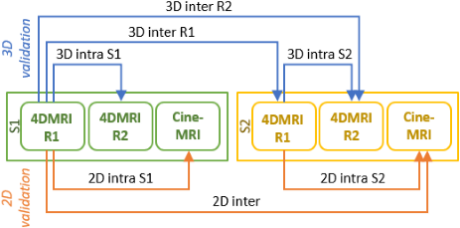
MRI‐based validation framework. Two 4DMRI (R1 and R2) and a cine‐MRI scan were acquired during the first (S1) and second (S2) session. These were used for 3D and 2D intra‐ and inter‐fraction motion testing. Arrows point from the building to the testing data set and labels indicates the corresponding test.

The respiratory motion model is built on the first‐acquired RC 4DMRI (R1) and tested on successively acquired data sets, considered as ground truth for model evaluation. This means that, for each testing data set, the corresponding surrogate is used to feed the motion model, and the resulting model estimates are compared against the ground truth data. Only a 2D comparison between the ground truth 2D cine‐MRI and the corresponding slice of the model estimate is viable (2D validation), whereas a 3D comparison is possible between the ground truth respiratory phases of the testing RC 4DMRI and the model estimates (3D validation). The evaluated scenarios are described in Table 1 and Figure 1.

**Table 1:** Summary of the acronyms used for the evaluated scenarios

Scenario	Type of evaluation	Data used	Aim of the test
3D intra‐S1 & 3D intra‐S2	3D	Model building: 4DMRI R1 (session 1 or session 2) Model testing: 4DMRI R2 (session 1 or session 2)	To account for intra‐fraction variations in session 1 and session 2
2D intra‐S1 & 2D intra‐S2	2D	Model building: 4DMRI R1 (session 1 or session 2) Model testing: Cine‐MRI (session 1 or session 2)	To account for intra‐fraction (i.e. cycle‐to‐cycle) variations in session 1 and session 2
3D inter‐R1 & 3D inter‐R2	3D	Model building: 4DMRI R1 (session 1) Model testing: 4DMRI R1 or R2 (session 2)	To account for inter‐fraction variations between session 1 and session 2
2D inter	2D	Model building: 4DMRI R1 (session 1) Model testing: Cine‐MRI (session 2)	To account for inter‐fraction variations between session 1 and session 2

### MRI data acquisition

**Fig. 2:**
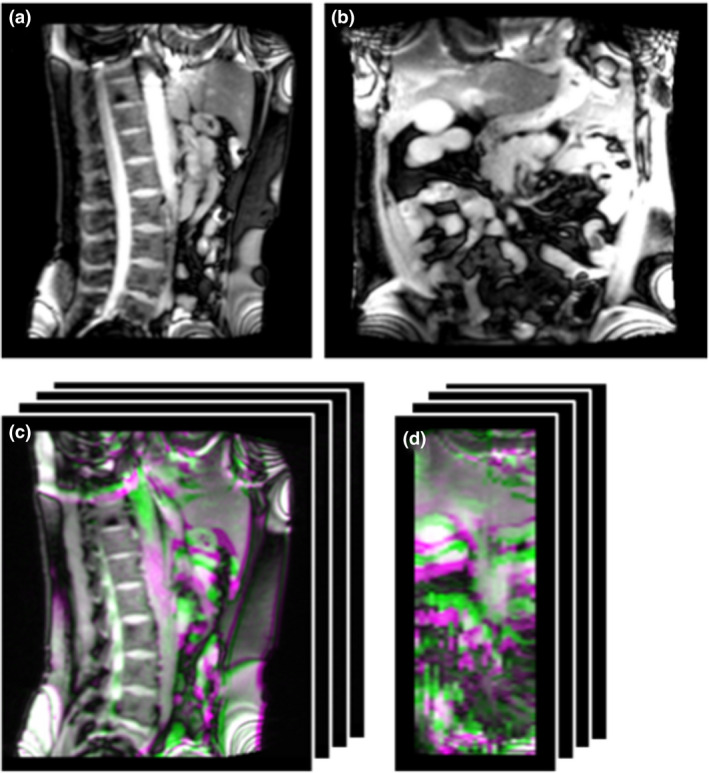
Example of acquired data (P02): sagittal (a) and coronal (b) cine‐MRI frames; sagittal (c) and coronal (d) view of the overlay between the end‐exhale (green) and end‐inhale (violet) phases of the 4DMRI.

Data from 5 pancreas (P02, P03, P04, P06, P07) and 2 liver patients (P01, P05) were acquired at the National Centre for Oncological Hadrontherapy (CNAO, Pavia, Italy)^22^, ^23^ with a Siemens Magnetom Verio 3T scanner. Patients underwent two acquisition sessions as explained in section 2.1; in particular, one was before treatment, the second approximately 1 week after the first scan. During the first MRI scan, interleaved sagittal/coronal 2D cine‐MR images centred on the tumour (Fig. 2a,b) were acquired in free breathing (TrueFISP sequence; pixel spacing: 1.33 × 1.33 mm; slice thickness: 5 mm; repetition time/echo time: 228.07 ms/1.5 ms; accelerating factor: 2; acquisition time: 230 ms). Additionally, multi‐slice sagittal images of the abdomen were acquired during free breathing with the same TrueFISP sequence and retrospectively sorted in a 4DMRI,^9^ comprising eight bins (breathing phases) and a field of view limited to 12.5 cm slab of sagittal slices centred in the tumour (Fig. 2c,d). It has to be noted that 4DMRI reconstruction artefacts due to the chosen retrospective sorting technique were quantified below 2 mm,^9^ that is below the maximum voxel size; thus, their impact on respiratory motion model validation is negligible. Also, the banding artefacts in the superior and inferior part of the images (Fig. 2) are expected not to impact the description of motion in the tumour area. As anticipated, repeated 4DMRI were collected within and between two sessions to evaluate the model performance in case of intra‐ and inter‐fraction motion. For patients P01, P02 and P03, only one 4DMRI for each session was acquired, whereas a single acquisition session was available for P07 because of patient positioning issues. For P01, it was not possible to evaluate the 2D inter‐fraction scenario due to the failure in obtaining the image‐based surrogate signal for motion model application.

### Respiratory motion model

The considered respiratory motion model^20^ (schematic in Appendix S1) relies on DIR to extract deformation vector fields (DVFs) describing the motion between a reference MRI – which corresponds to the end‐exhale MRI^21^ – and all other respiratory phases within the 4DMRI data set. In this study, DIR was performed through the multiresolution B‐splines algorithm available in Plastimatch (https://plastimatch.org/), and its error was evaluated^18^, ^19^ as it affects model estimations and represents its minimum uncertainty.^24^ A DVF describing the baseline variation was used to update the reference image^20^ in case of inter‐fraction motion: this DVF was obtained by registering the reference MRI (of the building 4DMRI) to the end‐exhale MRI of the testing data set (i.e. second 4DMRI of the first session or 4DMRI of the second session). The phase and amplitude values of each testing respiratory state, as extracted from an image‐based surrogate signal derived from 2D cine‐MRI planes (details in Appendix S2), were then used to interpolate the set of previously obtained DVFs and to scale the interpolated DVF, respectively. The updated reference volume was finally warped according to the estimated DVF, hence generating the 3D MRI volume representing the desired respiratory state.

### Respiratory motion model validation

#### DIR validation

The DIR error affecting the motion model performance was evaluated in a patient‐specific fashion.^19^ Specifically, the DIR between 4DMRI R1 phases of both S1 and S2 (4DMRI S1 DIR and 4DMRI S2 DIR, respectively) and between end‐exhale MRI volumes for baseline DVF estimation (INTER DIR) were evaluated. For 4DMRI DIR, only the end‐inhale respiratory phase was considered, as it is expected to correspond to the largest error. The scale‐invariant feature transform (SIFT)^25^, ^26^ was used to automatically extract points in the reference and registered images, and the 3D distance between corresponding points was computed.

#### 3D model validation with 4DMRI

For the 3D validation, the 3D MRIs estimated by the model were compared against the corresponding volumes of the (ground truth) testing 4DMRI. A global evaluation was performed by automatically extracting corresponding points in the estimated and the ground truth volumes by means of the SIFT^25^, ^26^ and computing their distance. An additional local evaluation was performed on the tumour contour, which was warped on both 4DMRIs from the reference 3DMRI. Specifically, the 3D distance between tumour centres of mass (COM) in corresponding phases was evaluated. The SIFT point distance and tumour COM distance were computed also between the testing end‐inhale MRI volumes and the reference end‐exhale MRI, in order to quantify the range of motion to be compensated by the model.

#### 2D model validation with 2D cine‐MRI

For cine‐MRI, only a 2D evaluation of the model performance was possible on coronal and sagittal planes. Specifically, the slices of the estimated 3D MRI (referred to as estimated slice) corresponding to the (ground truth) coronal/sagittal cine‐MRI frame were considered. Anatomical landmarks manually identified on the first cine‐MRI frames (Appendix S3) were tracked through template matching on all cine‐MRI frames and on the corresponding estimated slices, as well as on the end‐exhale and end‐inhale MRI slices of the building 4DMRI. In the 2D validation, the SIFT was not used due to poor performance on the estimated coronal slice. Then, the model performance was investigated by considering:

1. the distance between landmarks in corresponding slices, quantifying the estimation error (cine‐error);
2. the maximum displacement of cine‐MRI landmarks with respect to the end‐exhale MRI (which was used as reference for model building), thus evaluating respiratory motion during cine‐MRI acquisition (cine‐motion);
3. the landmarks range of motion in the building 4DMRI, for comparison with cine‐MRI motion (4DMRI‐motion).

## Results

### DIR validation

**Fig. 3:**
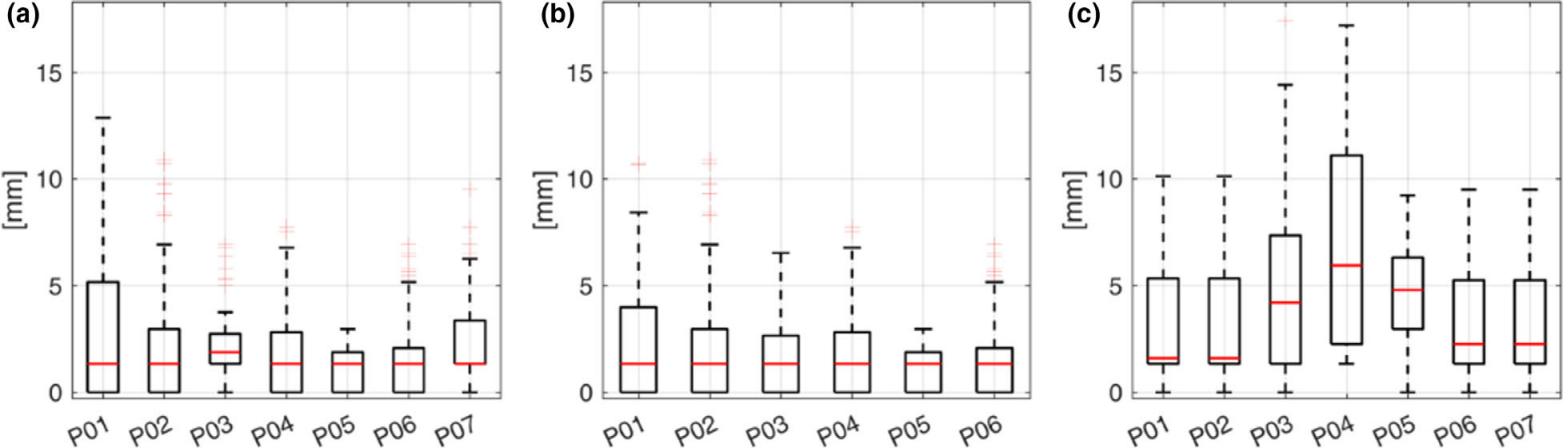
DIR error for 4DMRI S1 (a), 4DMRI S2 (b) and baseline (c) registrations.

Overall, the median (interquartile range) error was 1.33 (2.97) mm, 1.33 (2.66) mm and 2.97 (4.41) mm for the 4DMRI S1, 4DMRI S2 and INTER DIR, respectively. For 4DMRI S1 and 4DMRI S2 DIR, the median error was below 1.88 mm in all patients (Fig. 3a,b), whereas for INTER DIR the median error showed slightly larger values (Fig. 3c). For P04, the INTER DIR median (interquartile range) error was 5.95 (8.84) mm, since large inter‐fraction anatomical variations of the abdominal organs (mainly bowel) were found (8.40 mm mean amplitude), and DIR did not totally compensate for them (Appendix S4).

### 3D model validation with 4DMRI

**Fig. 4:**
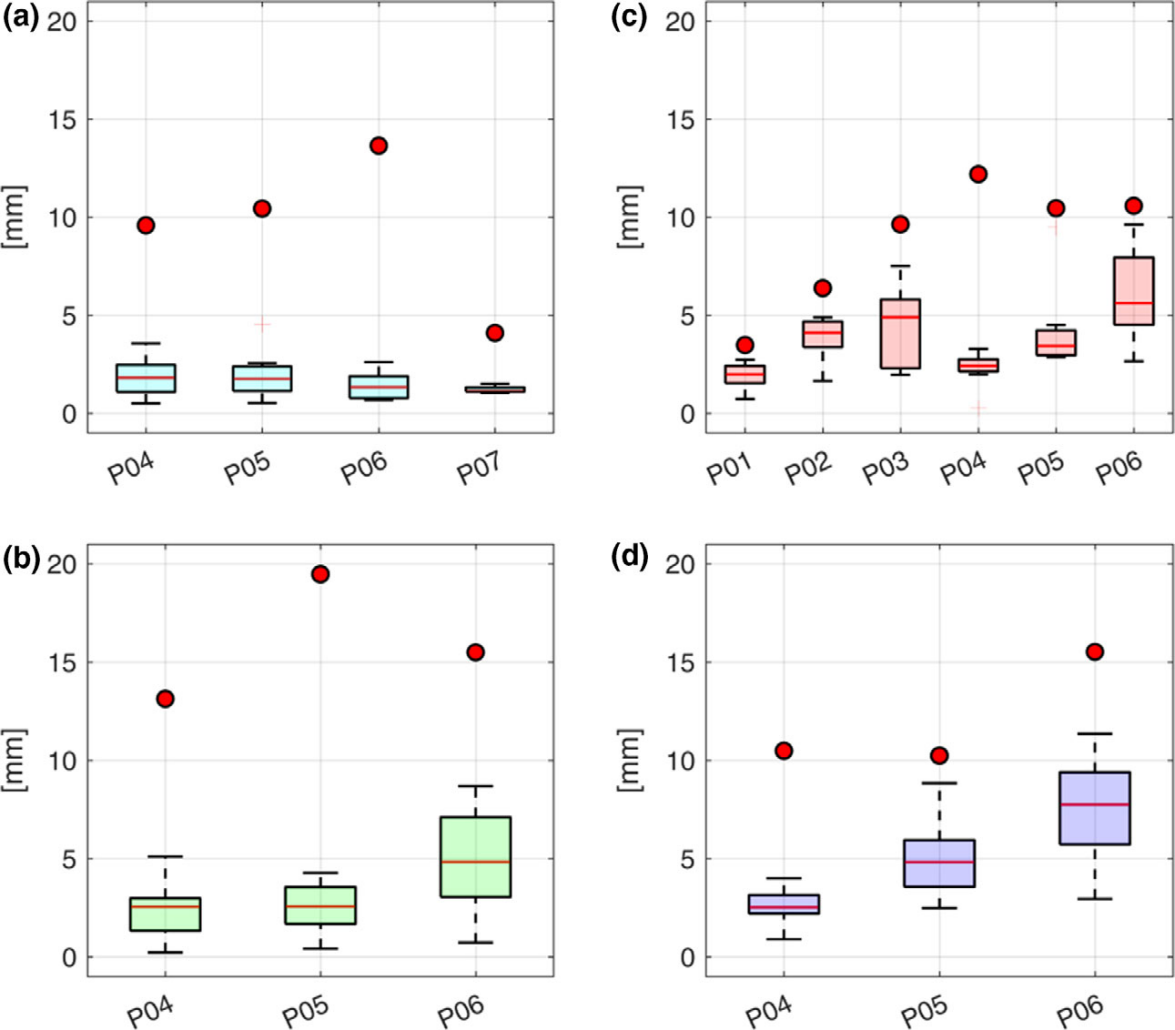
Boxplots and circles represent the model estimation error and the range of motion to be compensated for by the model, respectively, as quantified by tumour COM distances in the (a) 3D intra‐S1, (b) 3D intra‐S2, (c) 3D inter‐R1 and (d) 3D inter‐R2 scenarios.

The error on corresponding points between the estimated and ground truth volumes, as well as the errors in terms of COM, reflected the trend observed on DIR error. Specifically, the error on corresponding points resulted in median of 1.33 mm over all cases when testing intra‐fraction motion variations (3D intra‐S1 and 3D intra‐S2), with a tumour COM error in median of 1.81 mm, whereas slightly higher errors (4.21 mm and 3.47 mm for corresponding points and tumour COM, respectively) were quantified in the presence of inter‐fraction variations (3D inter‐R1 and 3D inter‐R2). Nevertheless, for all the investigated scenarios, the estimation error was below the range of motion to be estimated by the model (Table 2 and Fig. 4).

**Table 2:** Results of the 3D validation: the estimation error was computed between ground truth and estimated volumes, the range of motion to be compensated by the model was quantified between the end‐exhale and end‐inhale phases of the ground truth 4DMRI. Median (interquartile range) results over all patients are reported for each scenario

	3D intra‐S1		3D intra‐S2		3D inter‐R1		3D inter‐R2	
	Estimation error [mm]	Range of motion [mm]	Estimation error [mm]	Range of motion [mm]	Estimation error [mm]	Range of motion [mm]	Estimation error [mm]	Range of motion [mm]
SIFT points distance	1.33 (1.88)	4.20 (7.10)	1.88 (2.86)	5.82 (8.77)	4.20 (4.65)	6.93 (9.60)	4.50 (4.83)	5.82 (13.87)
Tumour COM distance	1.33 (1.04)	10.01 (5.20)	2.91 (2.43)	15.50 (4.76)	3.11 (2.47)	10.05 (4.19)	4.27 (3.79)	10.49 (3.96)

### 2D model validation with 2D cine‐MRI

Overall, the median cine‐error was 3.76 mm, 2.97 mm and 3.99 mm for the 2D intra‐S1, 2D intra‐S2 and 2D inter‐scenario, respectively (Table 3). The quantified cine‐motion was comparable to the 4DMRI‐motion in all the three scenarios. The median estimation error ranged between 1.88 mm and 5.32 mm (qualitative results related to P06, associated with the largest median error, are represented in Appendix S5), whereas the median respiratory motion was in the range 2.97 mm to 14.32 mm in the cine‐MRI and between 2.97 mm and 13.89 mm in the building 4DMRI (Table 3).

**Table 3:** Results of 2D respiratory motion model validation with cine‐MRI data. The median (interquartile range) values among all landmarks and all patients are reported

Patient	2D intra‐S1			2D intra‐S2			2D inter		
	cine‐error [mm]	cine‐motion [mm]	4DMRI‐motion [mm]	cine‐error [mm]	cine‐motion [mm]	4DMRI‐motion [mm]	cine‐error [mm]	cine‐motion [mm]	4DMRI‐motion [mm]
P01	4.21 (3.99)	2.97 (1.88)	2.97 (1.88)	4.21 (4.12)	8.41 (2.86)	8.41 (2.86)	‐	‐	‐
P02	3.76 (5.87)	5.48 (3.15)	4.21 (3.99)	2.97 (3.47)	6.65 (4.21)	8.41 (4.13)	3.99 (2.98)	6.65 (4.21)	4.21 (3.99)
P03	2.66 (2.43)	9.52 (9.47)	5.48 (9.52)	1.88 (1.64)	4.76 (1.28)	3.99 (1.37)	2.97 (2.32)	4.76 (1.28)	5.48 (9.52)
P04	3.99 (2.51)	11.97 (2.46)	10.72 (3.08)	3.99 (2.82)	12.04 (3.17)	8.41 (5.86)	3.99 (3.29)	12.04 (3.17)	10.72 (3.08)
P05	4.80 (3.99)	14.32 (5.02)	13.89 (5.92)	2.97 (5.28)	11.90 (3.24)	14.32 (4.94)	4.21 (3.68)	11.90 (3.24)	13.89 (5.92)
P06	3.76 (2.66)	11.97 (7.13)	12.77 (6.59)	4.80 (2.67)	8.58 (3.44)	13.56 (4.84)	5.32 (5.95)	8.58 (3.44)	12.77 (6.59)
P07	4.10 (6.65)	11.97 (12.21)	11.97 (12.21)	–	–	–	–	–	–
	3.76 (4.07)	10.72 (9.68)	11.97 (8.84)	2.97 (3.60)	8.41 (8.77)	8.52 (6.40)	3.99 (3.29)	10.97 (8.72)	9.40 (6.50)

## Discussion

In this study, a validation framework based on MRI data of patients with abdominal tumours was established to test a global respiratory motion model ^20^ in the presence of breathing motion variabilities. The main advantages of the framework are that (i) it uses MRI, which is an ionizing radiation‐free imaging modality, thus avoiding non‐therapeutic dose to the patients, and (ii) it provides a clinical ground truth for motion model evaluation by means of repeated acquisition sessions, thus accounting for imaging non‐idealities and realism of anatomy variability that are only partially represented in computational phantoms’ studies.^27^ The framework is therefore useful to assess, prior to the clinical application of a certain respiratory motion model in an MRI‐guided workflow, the accuracy and limitations of the modelling tool. The framework entails the use of both TR and RC (4D) MRI modalities allowing the evaluation of cycle‐to‐cycle and intra‐/inter‐fraction variability, respectively.

The considered motion model relies on DIR for model building and inter‐fraction baseline compensation; therefore, the DIR error was quantified^18^, ^19^ and demonstrated to be comparable to the voxel size. Slightly higher DIR errors were quantified in case of large variations caused by inter‐fraction motion in the abdomen (2.97 mm vs. 1.33 mm median value for inter‐fraction baseline DIR vs. DIR between 4DMRI respiratory phases, respectively).

As such, concerning the 3D motion model validation, the estimation error was higher in the inter‐fraction scenario with respect to the intra‐fraction one (4.21 mm vs. 1.33 mm median values, respectively), as clear from Fig. 4. This was mainly caused by inter‐fraction variations of the abdominal organs (e.g. filling of stomach and intestine) which were not perfectly compensated for by the DIR. The 2D validation showed that the investigated global motion model was able to estimate cycle‐to‐cycle variations as depicted by 2D cine‐MRI with a median error comparable to the voxel size and below the motion described in both the testing cine‐MRI and building 4DMRI (Table 3). The Jacobian determinant of the estimated DVF^18^ was also analysed, resulting non‐negative in more than 99% of the volume and thus demonstrating the physical plausibility of estimated deformations (Appendix S6).

The proposed validation framework confirms that the global motion model performance is dependent on DIR accuracy, which therefore requires a patient‐specific evaluation,^19^ and that the model shows proper estimations of breathing motion in the imaged site when intra‐fraction and cycle‐to‐cycle variations occurred. The model, however, presents poorer accuracy when considering inter‐fraction baseline variations, as found in similar studies,^28^ so that a new MRI data acquisition for building of the model rather than a baseline update is recommended between radiotherapy fractions for the investigated anatomical sites.

One limitation of the study consists in the restricted field of view of the 4DMRI (12.5 cm in the right‐left direction), which was due to imaging constraints and the trade‐off between temporal and spatial resolution.^9^, ^29^ The availability of 4DMRI data with extended field of view^7^, ^30^ would lead to better DIR performance and improved motion model estimations over the entire irradiated patient anatomy. Another limitation is the small number of patient data sets acquired for our analysis. This was due to recruitment issues and technical problems, mainly related to patients’ compliance and MR artefacts due to the presence of air in the stomach and the bowel. Nevertheless, the proposed MRI‐based validation framework can be easily extended in the future to a larger patient cohort and used for the patient‐specific validation of alternative global respiratory motion models^27^ in conjunction with, or as support of, current approaches based on phantoms.

In conclusion, the acquisition of 2D cine‐MRI and repeated 4DMRI on patients with abdominal tumours allowed us to provide the groundwork for the definition of a validation framework that is put forward to create the required imaging data set for the evaluation of global motion models in MRI‐guided treatments.

## Supporting information


**Appendix S1.** Respiratory motion model schematic.
**Appendix S2.** Phase and amplitude estimation.
**Appendix S3.** Manually identified landmarks.
**Appendix S4.** Deformable Image Registration validation.
**Appendix S5.** Qualitative results.
**Appendix S6.** Jacobian analysis of model output.
