# Supplementary material for: An MRI framework for respiratory motion modelling validation
Source: J Med Imaging Radiat Oncol. 2021 Mar 26;65(3):337–44. doi: 10.1111/1754-9485.13175 (PMC8251859; doi:10.1111/1754-9485.13175)
Supplement: Supplementary file 1 — Appendix S1. Respiratory motion model schematic. Appendix S2. Phase and amplitude estimation. Appendix S3. Manually identified landmarks. Appendix S4. Deformable Image Registration validation. Appendix S5. Qualitative results. Appendix S6. Jacobian analysis of model output. [file ARA-65-337-s001.pdf]

Appendix S1. Respiratory motion model schematic

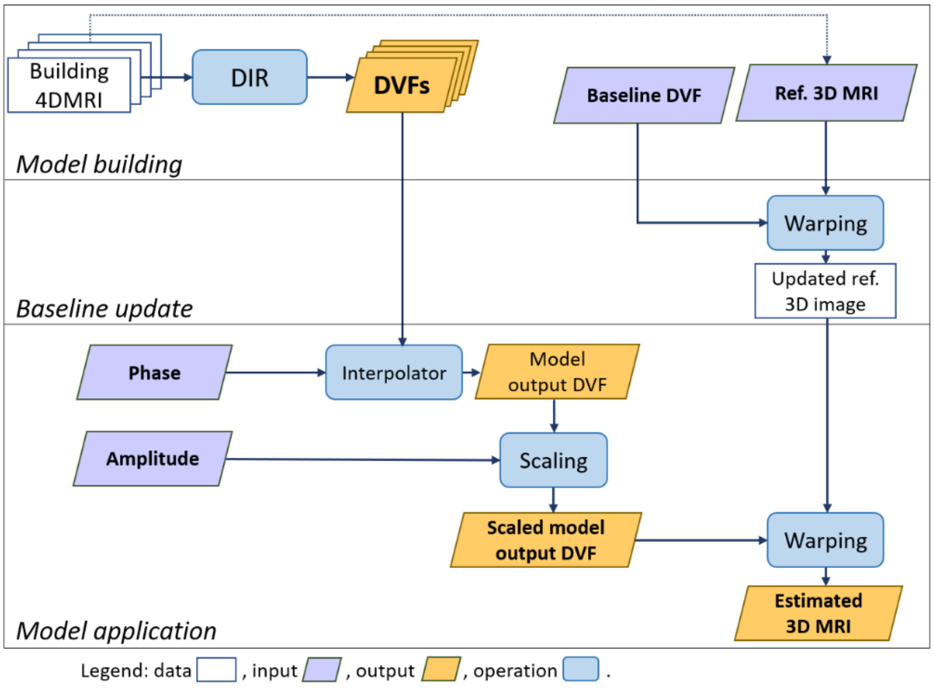

Figure s1. Respiratory motion model schematic: during model building, deformable image registration (DIR) is applied on the 4DMRI dataset to obtain a set of deformable vector fields (DVFs), the reference MRI and the baseline DVF are considered; for the baseline update, the reference (ref.) MRI is warped according to the baseline DVF; during model application, the specified phase and amplitude values are used to interpolate the set of DVFs and scale the interpolated DVF, respectively; finally the updated reference image is warped (i.e. deformed) according to the obtained DVF, thus estimating the MRI volume.

Appendix S2. Phase and amplitude estimation

To derive phase and amplitude values to feed the motion model, an image-based mono-dimensional surrogate was obtained. The sagittal and coronal planes corresponding to the cine-MRI slices were extracted from the building and testing 4DMRI volumes. Corresponding anatomical points were automatically extracted between the building end-exhale MRI slices and the first couple of frames of the cine-MRI by means of the Scale-Invariant Feature Transform (SIFT [25,26]). These points were tracked on other phases from the building and testing 4DMRI datasets and on all subsequent cine-MRI frames through template matching. Therefore, each time point was described by the coordinates of the selected points on the couple of sagittal and coronal frames from the respective dataset.

All the 4DMRI were reconstructed with the same number of volumes, therefore the phase values of the testing 4DMRI volumes always corresponded to the phase values of the building 4DMR. Differently, for cine-MRI, the Principal Component Analysis (PCA) on the points' coordinates was performed, and the first PC was considered. The peaks (end-inhale) and valleys (end-exhale) were identified and the phase was linearly interpolated between them.

The amplitude parameter was computed as the ratio between the mean points motion (with respect to end-exhale) in the testing data and in the corresponding phase of the building 4DMRI.

Appendix S3. Manually identified landmarks

Due to poor performance of SIFT algorithm in identifying corresponding points between the coronal cine-MRI and the estimated coronal slice in the reference volume, ten anatomical landmarks were manually selected on the first coronal and sagittal cine-MRI frames (Figure s2). Then, the landmarks were tracked through template matching on all cine-MRI frames. To check for consistent tracking, the correlation between the resulting trajectories was evaluated: poor correlation to other trajectories indicated limited performance of the tracking[1], therefore the corresponding points were discarded. The group of points presenting maximum mutual correlation between trajectories were considered for the 2D validation (mean number of points: 8.2).

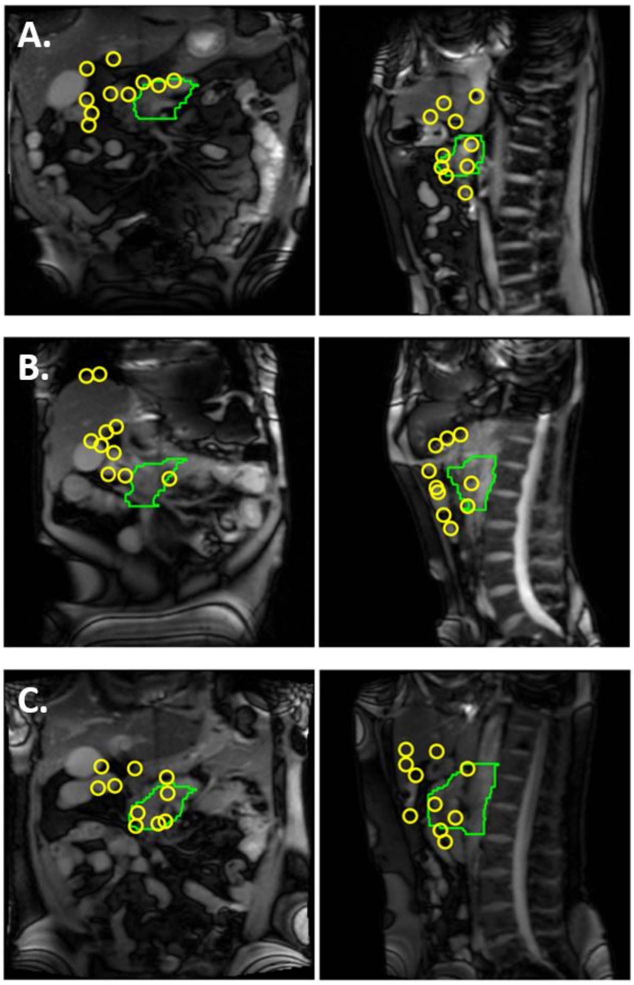

Figure s2. Coronal (left column) and sagittal (right column) cine-MRI frames with Clinical Target Volume contour (green) and selected anatomical landmarks (yellow circles). A. P02, B. P03, C. P04.

[1] Meschini et al. 2020 Improvement of retrospective sorting for artefact reduction in 4DMRI of the abdominal site (PTCOG58-0279) DOI 10.14338/IJPT.19-PTCOG-6.4

Appendix S4. Deformable Image Registration validation

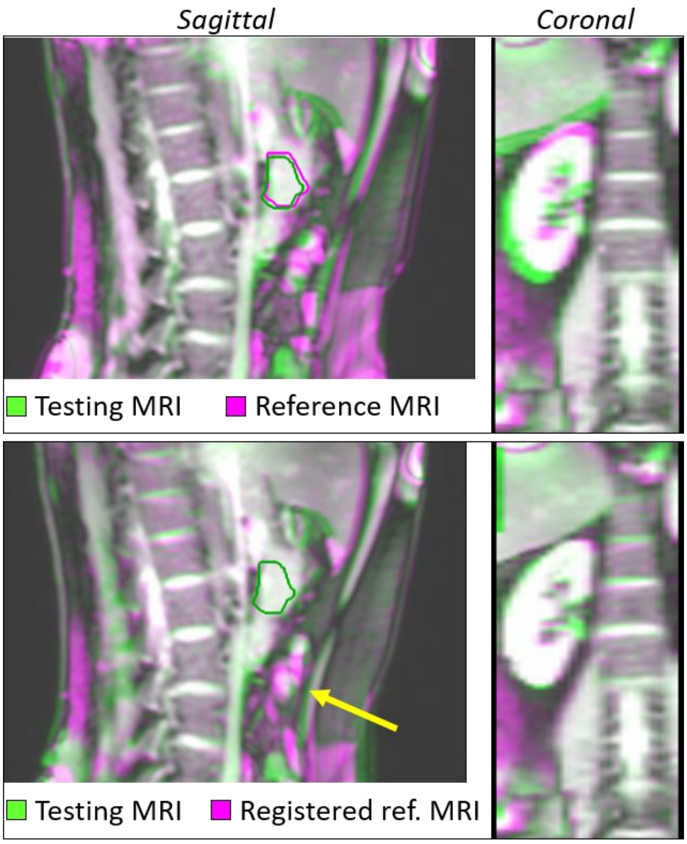

Figure s3. Inter-fraction variations for P04 (upper panel) and DIR error after baseline DVF application (bottom panel). The yellow arrow highlights DIR inaccuracy in the bowel.

Appendix S5. Qualitative results

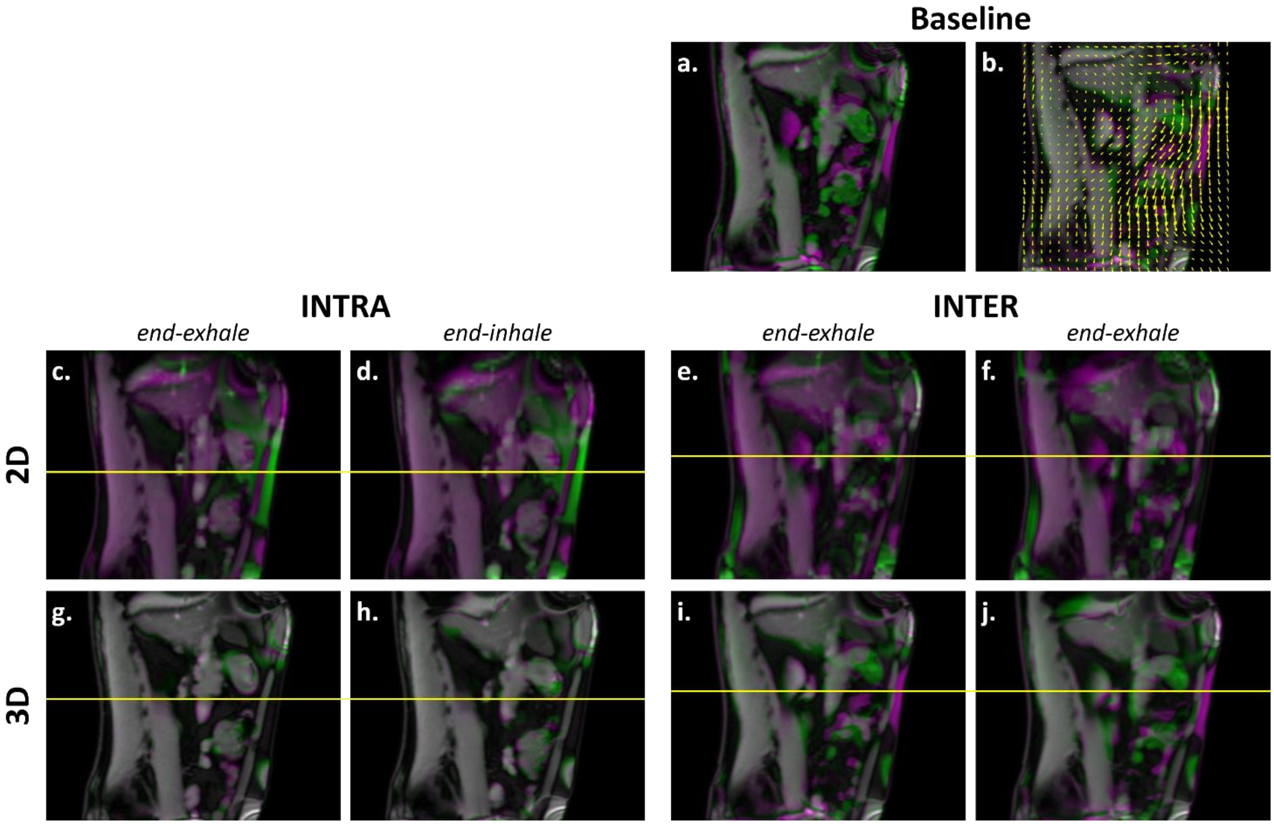

Figure s4. Qualitative results for P06. Baseline variations: (a.) overlay between the reference MRI and the end-exhale phase of 4DMRI R1 S2; (b.) baseline DIR output. Model estimation error: overlay between model output (violet) and GT slices (green), for the 2D intra S1 (c.-d.), 2D inter (e.-f.), 3D intra S1 (g.-h.), 3D inter R1 (i.-j.) scenarios. The yellow line highlights respiratory motion between the end-exhale (left) and end-inhale (right) respiratory phases in each scenario.

Appendix S6. Jacobian analysis of model output

To verify that the vector fields estimated by the respiratory motion model represent anatomically consistent deformations, the Jacobian of the vector fields [18] was evaluated. Specifically, for each patient we considered the vector field corresponding to the end-inhale respiratory phase with the maximum computed amplitude, which corresponds to the maximum deformation estimated by the model. Then, we computed the percentage of negative voxels in the Jacobian of the vector field (referred to as $p_{J<0}$), which indicate a physically non-plausible deformation.

For all patients, the $p_{J<0}$ was below 1%, as seen in Table s1.

Table s1. Percentage of negative voxels in the Jacobian of the maximum estimated vector field.

**Patient**	$\mathbf{p_{J<0}}$
P01	0.04%
P02	0.00%
P03	0.00%
P04	0.09%
P05	0.49%
P06	0.08%
P07	0.02%
